# Supplementary material for: Phylogenetic analysis of SARS-CoV-2 lineage development across the first and second waves in Eastern Germany in 2020: insights into the cause of the second wave
Source: Epidemiol Infect. 2021 Jul 30;149:e177. doi: 10.1017/S0950268821001461 (PMC8365105; doi:10.1017/S0950268821001461)
Supplement: Supplementary file 1 [file S0950268821001461sup001.doc]

**Supplementary Table S1. Detailed information of SARS-CoV-2 genome sequences from Eastern Germany, March to December, 2020.**

| **Strain** | **Country** | **Admin Division** | **gisaid_epi_isl** | **Collection Date** |
| --- | --- | --- | --- | --- |
| **hCoV-19/Germany/BE-ChVir-W1317-7453/2020** | Germany | Berlin | EPI_ISL_753746 | 2020-04-26 |
| **hCoV-19/Germany/BE-ChVir1897/2020** | Germany | Berlin | EPI_ISL_729482 | 2020-03-17 |
| **hCoV-19/Germany/BE-ChVir2192/2020** | Germany | Berlin | EPI_ISL_729499 | 2020-03-05 |
| **hCoV-19/Germany/BE-ChVir-D900-8163/2020** | Germany | Berlin | EPI_ISL_753799 | 2020-03-15 |
| **hCoV-19/Germany/BB-ChVir-D936-7299/2020** | Germany | Brandenburg | EPI_ISL_753722 | 2020-03-13 |
| **hCoV-19/Germany/BB-ChVir-D935-7207/2020** | Germany | Brandenburg | EPI_ISL_753814 | 2020-03-13 |
| **hCoV-19/Germany/BE-ChVir-D716-2070/2020** | Germany | Berlin | EPI_ISL_753822 | 2020-03-05 |
| **hCoV-19/Germany/BB-ChVir-W1041-5644/2020** | Germany | Brandenburg | EPI_ISL_753843 | 2020-03-25 |
| **hCoV-19/Germany/BE-ChVir-W1081-8197/2020** | Germany | Berlin | EPI_ISL_753933 | 2020-04-06 |
| **hCoV-19/Germany/BE-ChVir-W1143-8140/2020** | Germany | Berlin | EPI_ISL_753732 | 2020-04-08 |
| **hCoV-19/Germany/ST-MD012/2020** | Germany | Saxony-Anhalt | EPI_ISL_728348 | 2020-04-16 |
| **hCoV-19/Germany/ST-MD019/2020** | Germany | Saxony-Anhalt | EPI_ISL_728351 | 2020-04-16 |
| **hCoV-19/Germany/ST-MD030/2020** | Germany | Saxony-Anhalt | EPI_ISL_728354 | 2020-04-16 |
| **hCoV-19/Germany/ST-MD006/2020** | Germany | Saxony-Anhalt | EPI_ISL_728346 | 2020-04-16 |
| **hCoV-19/Germany/ST-z4/2020** | Germany | Saxony-Anhalt | EPI_ISL_776771 | 2020-04-16 |
| **hCoV-19/Germany/ST-z2/2020** | Germany | Saxony-Anhalt | EPI_ISL_776711 | 2020-04-16 |
| **hCoV-19/Germany/ST-MD037/2020** | Germany | Saxony-Anhalt | EPI_ISL_728356 | 2020-04-16 |
| **hCoV-19/Germany/ST-MD038/2020** | Germany | Saxony-Anhalt | EPI_ISL_728357 | 2020-04-16 |
| **hCoV-19/Germany/BB-ChVir3232/2020** | Germany | Brandenburg | EPI_ISL_729548 | 2020-03-31 |
| **hCoV-19/Germany/BE-RKI-Z-0007/2020** | Germany | Berlin | EPI_ISL_483142 | 2020-04-20 |
| **hCoV-19/Germany/BE-ChVir3087/2020** | Germany | Berlin | EPI_ISL_729404 | 2020-03-24 |
| **hCoV-19/Germany/BE-RKI-Z-0013/2020** | Germany | Berlin | EPI_ISL_483148 | 2020-04-27 |
| **hCoV-19/Germany/BE-RKI-Z-0006/2020** | Germany | Berlin | EPI_ISL_483141 | 2020-04-20 |
| **hCoV-19/Germany/BE-ChVir-W1072-4344/2020** | Germany | Berlin | EPI_ISL_753930 | 2020-04-02 |
| **hCoV-19/Germany/BE-ChVir-W1048-4346/2020** | Germany | Berlin | EPI_ISL_753926 | 2020-03-27 |
| **hCoV-19/Germany/SN-ChVir-W1275-4371/2020** | Germany | Saxony | EPI_ISL_754052 | 2020-04-15 |
| **hCoV-19/Germany/BE-ChVir-D751-5650/2020** | Germany | Berlin | EPI_ISL_754057 | 2020-03-13 |
| **hCoV-19/Germany/BB-ChVir-W1062-7296/2020** | Germany | Brandenburg | EPI_ISL_753846 | 2020-03-31 |
| **hCoV-19/Germany/BE-ChVir-3181-5746/2020** | Germany | Berlin | EPI_ISL_806541 | 2020-04-06 |
| **hCoV-19/Germany/BE-ChVir-D711-11/2020** | Germany | Berlin | EPI_ISL_516639 | 2020-03-07 |
| **hCoV-19/Germany/SN-DcGC-L62668/2020** | Germany | Saxony | EPI_ISL_887110 | 2020-09-29 |
| **hCoV-19/Germany/SN-DcGC-L62676/2020** | Germany | Saxony | EPI_ISL_887114 | 2020-10-05 |
| **hCoV-19/Germany/BE-ChVir-W1074-8144/2020** | Germany | Berlin | EPI_ISL_753931 | 2020-04-03 |
| **hCoV-19/Germany/BE-ChVir-W1082-8115/2020** | Germany | Berlin | EPI_ISL_753934 | 2020-04-05 |
| **hCoV-19/Germany/BE-ChVir1959/2020** | Germany | Berlin | EPI_ISL_729489 | 2020-03-07 |
| **hCoV-19/Germany/BE-ChVir-W1248-16/2020** | Germany | Berlin | EPI_ISL_516644 | 2020-03-07 |
| **hCoV-19/Germany/BE-ChVir-D715-D799-17/2020** | Germany | Berlin | EPI_ISL_516645 | 2020-03-06 |
| **hCoV-19/Germany/BE-ChVir-D754-8111/2020** | Germany | Berlin | EPI_ISL_754058 | 2020-03-16 |
| **hCoV-19/Germany/TH-IIMK-CaSe-2/2020** | Germany | Thuringia | EPI_ISL_728292 | 2020-04-03 |
| **hCoV-19/Germany/BE-RKI-Z-0004/2020** | Germany | Berlin | EPI_ISL_483139 | 2020-04-18 |
| **hCoV-19/Germany/BE-RKI-Z-0005/2020** | Germany | Berlin | EPI_ISL_483140 | 2020-04-18 |
| **hCoV-19/Germany/BE-ChVir1670/2020** | Germany | Berlin | EPI_ISL_729477 | 2020-03-02 |
| **hCoV-19/Germany/SN-DcGC-L56893/2020** | Germany | Saxony | EPI_ISL_887106 | 2020-04-09 |
| **hCoV-19/Germany/TH-IIMK-CaSe-20/2020** | Germany | Thuringia | EPI_ISL_763077 | 2020-03-20 |
| **hCoV-19/Germany/BE-RKI-Z-0018/2020** | Germany | Berlin | EPI_ISL_483152 | 2020-04-20 |
| **hCoV-19/Germany/BE-ChVir-W1281-8144/2020** | Germany | Berlin | EPI_ISL_753946 | 2020-04-16 |
| **hCoV-19/Germany/BE-ChVir-D762-6625/2020** | Germany | Berlin | EPI_ISL_753918 | 2020-03-13 |
| **hCoV-19/Germany/BE-ChVir-W1726-8250/2020** | Germany | Berlin | EPI_ISL_754013 | 2020-08-20 |
| **hCoV-19/Germany/BE-ChVir-D1937-8281/2020** | Germany | Berlin | EPI_ISL_753731 | 2020-09-25 |
| **hCoV-19/Germany/BE-ChVir-D2022-4412/2020** | Germany | Berlin | EPI_ISL_753905 | 2020-10-10 |
| **hCoV-19/Germany/BE-ChVir-D1979-4481/2020** | Germany | Berlin | EPI_ISL_753818 | 2020-10-09 |
| **hCoV-19/Germany/BE-ChVir-W1715-6702/2020** | Germany | Berlin | EPI_ISL_754005 | 2020-08-08 |
| **hCoV-19/Germany/BE-ChVir-D2049-8234/2020** | Germany | Berlin | EPI_ISL_753716 | 2020-10-05 |
| **hCoV-19/Germany/BE-ChVir-W1692-8235/2020** | Germany | Berlin | EPI_ISL_753987 | 2020-07-08 |
| **hCoV-19/Germany/BE-ChVir-W1647-6745/2020** | Germany | Berlin | EPI_ISL_753980 | 2020-08-02 |
| **hCoV-19/Germany/TH-IIMK-CaSe-1/2020** | Germany | Thuringia | EPI_ISL_632938 | 2020-04-19 |
| **hCoV-19/Germany/TH-IIMK-CaSe-11/2020** | Germany | Thuringia | EPI_ISL_728323 | 2020-08-18 |
| **hCoV-19/Germany/BE-ChVir-D1920-8202/2020** | Germany | Berlin | EPI_ISL_753866 | 2020-10-05 |
| **hCoV-19/Germany/BE-ChVir-D2007-8200/2020** | Germany | Berlin | EPI_ISL_753896 | 2020-10-15 |
| **hCoV-19/Germany/BE-ChVir-D1998-4426/2020** | Germany | Berlin | EPI_ISL_753890 | 2020-10-09 |
| **hCoV-19/Germany/BE-ChVir-D2020-4418/2020** | Germany | Berlin | EPI_ISL_753903 | 2020-10-11 |
| **hCoV-19/Germany/BE-ChVir-D2010-8268/2020** | Germany | Berlin | EPI_ISL_753898 | 2020-10-15 |
| **hCoV-19/Germany/BE-ChVir-D1999-8262/2020** | Germany | Berlin | EPI_ISL_753891 | 2020-10-13 |
| **hCoV-19/Germany/BB-RKI-NP-0077/2020** | Germany | Brandenburg | EPI_ISL_763052 | 2020-12-21 |
| **hCoV-19/Germany/TH-RKI-NP-0074/2020** | Germany | Thuringia | EPI_ISL_762999 | 2020-12-21 |
| **hCoV-19/Germany/BE-ChVir-D1958-8276/2020** | Germany | Berlin | EPI_ISL_753886 | 2020-09-28 |
| **hCoV-19/Germany/BB-RKI-NP-0036/2020** | Germany | Brandenburg | EPI_ISL_763060 | 2020-12-09 |
| **hCoV-19/Germany/ST-MD231/2020** | Germany | Saxony-Anhalt | EPI_ISL_894163 | 2020-12-26 |
| **hCoV-19/Germany/ST-MD261/2020** | Germany | Saxony-Anhalt | EPI_ISL_884286 | 2020-12-29 |
| **hCoV-19/Germany/ST-MD242/2020** | Germany | Saxony-Anhalt | EPI_ISL_884270 | 2020-12-29 |
| **hCoV-19/Germany/SN-RKI-NP-0079/2020** | Germany | Saxony | EPI_ISL_860298 | 2020-12-28 |
| **hCoV-19/Germany/ST-MD183/2020** | Germany | Saxony-Anhalt | EPI_ISL_864575 | 2020-10-28 |
| **hCoV-19/Germany/ST-MD204/2020** | Germany | Saxony-Anhalt | EPI_ISL_894164 | 2020-12-21 |
| **hCoV-19/Germany/BE-ChVir-W1080-4357/2020** | Germany | Berlin | EPI_ISL_753754 | 2020-04-05 |
| **hCoV-19/Germany/BE-ChVir-W1139-8123/2020** | Germany | Berlin | EPI_ISL_753940 | 2020-04-06 |
| **hCoV-19/Germany/BE-ChVir-W1446-8293/2020** | Germany | Berlin | EPI_ISL_753747 | 2020-06-24 |
| **hCoV-19/Germany/BE-ChVir-W1417-8240/2020** | Germany | Berlin | EPI_ISL_753959 | 2020-06-22 |
| **hCoV-19/Germany/BE-ChVir-W1719-6702/2020** | Germany | Berlin | EPI_ISL_754008 | 2020-08-16 |
| **hCoV-19/Germany/BE-ChVir-W1714-6785/2020** | Germany | Berlin | EPI_ISL_754004 | 2020-08-06 |
| **hCoV-19/Germany/BE-ChVir-D1926-5089/2020** | Germany | Berlin | EPI_ISL_753870 | 2020-10-10 |
| **hCoV-19/Germany/BE-ChVir-D1922-4497/2020** | Germany | Berlin | EPI_ISL_753867 | 2020-10-05 |
| **hCoV-19/Germany/BE-ChVir-D1978-4478/2020** | Germany | Berlin | EPI_ISL_753817 | 2020-10-10 |
| **hCoV-19/Germany/BE-ChVir-D2015-8253/2020** | Germany | Berlin | EPI_ISL_753776 | 2020-10-16 |
| **hCoV-19/Germany/BE-ChVir-D2035-8263/2020** | Germany | Berlin | EPI_ISL_753913 | 2020-10-09 |
| **hCoV-19/Germany/BE-ChVir-D2037-8226/2020** | Germany | Berlin | EPI_ISL_753915 | 2020-10-09 |
| **hCoV-19/Germany/BE-ChVir-W1543-4324/2020** | Germany | Berlin | EPI_ISL_753749 | 2020-06-22 |
| **hCoV-19/Germany/BE-ChVir-W1645-6781/2020** | Germany | Berlin | EPI_ISL_753979 | 2020-08-05 |
| **hCoV-19/Germany/BE-ChVir-W1635-6790/2020** | Germany | Berlin | EPI_ISL_753973 | 2020-08-05 |
| **hCoV-19/Germany/BE-ChVir-W2123-4456/2020** | Germany | Berlin | EPI_ISL_754019 | 2020-10-16 |
| **hCoV-19/Germany/BE-ChVir9029/2020** | Germany | Berlin | EPI_ISL_729605 | 2020-09-20 |
| **hCoV-19/Germany/BE-ChVir9012/2020** | Germany | Berlin | EPI_ISL_729604 | 2020-09-22 |
| **hCoV-19/Germany/BB-ChVir9011/2020** | Germany | Brandenburg | EPI_ISL_729387 | 2020-09-23 |
| **hCoV-19/Germany/ST-MD110/2020** | Germany | Saxony-Anhalt | EPI_ISL_803874 | 2020-12-08 |
| **hCoV-19/Germany/BE-ChVir-W1638-6728/2020** | Germany | Berlin | EPI_ISL_753975 | 2020-08-02 |
| **hCoV-19/Germany/BE-ChVir-W1730-6753/2020** | Germany | Berlin | EPI_ISL_753734 | 2020-08-08 |
| **hCoV-19/Germany/ST-MD254/2020** | Germany | Saxony-Anhalt | EPI_ISL_884265 | 2020-12-31 |
| **hCoV-19/Germany/BE-ChVir-W1717-6729/2020** | Germany | Berlin | EPI_ISL_754007 | 2020-08-15 |
| **hCoV-19/Germany/ST-MD209/2020** | Germany | Saxony-Anhalt | EPI_ISL_884283 | 2020-12-23 |
| **hCoV-19/Germany/ST-MD217/2020** | Germany | Saxony-Anhalt | EPI_ISL_884280 | 2020-12-19 |
| **hCoV-19/Germany/ST-MD214/2020** | Germany | Saxony-Anhalt | EPI_ISL_894165 | 2020-12-20 |
| **hCoV-19/Germany/ST-MD215/2020** | Germany | Saxony-Anhalt | EPI_ISL_884282 | 2020-12-21 |
| **hCoV-19/Germany/BE-ChVir-W1285-4367/2020** | Germany | Berlin | EPI_ISL_753948 | 2020-04-18 |
| **hCoV-19/Germany/BE-ChVir-D1986-4419/2020** | Germany | Berlin | EPI_ISL_753762 | 2020-10-02 |
| **hCoV-19/Germany/BE-ChVir-D1950-4422/2020** | Germany | Berlin | EPI_ISL_753803 | 2020-10-05 |
| **hCoV-19/Germany/ST-MD250/2020** | Germany | Saxony-Anhalt | EPI_ISL_894168 | 2020-12-30 |
| **hCoV-19/Germany/BE-ChVir-D2013-8255/2020** | Germany | Berlin | EPI_ISL_753804 | 2020-10-19 |
| **hCoV-19/Germany/ST-MD253/2020** | Germany | Saxony-Anhalt | EPI_ISL_884269 | 2020-12-31 |
| **hCoV-19/Germany/BB-ChVir-D1973-8218/2020** | Germany | Brandenburg | EPI_ISL_753745 | 2020-10-05 |
| **hCoV-19/Germany/BE-ChVir-D1949-8257/2020** | Germany | Berlin | EPI_ISL_753802 | 2020-10-05 |
| **hCoV-19/Germany/SN-RKI-Z-0039/2020** | Germany | Saxony | EPI_ISL_825163 | 2020-12-23 |
| **hCoV-19/Germany/SN-RKI-Z-0040/2020** | Germany | Saxony | EPI_ISL_825164 | 2020-12-23 |
| **hCoV-19/Germany/TH-RKI-NP-0043/2020** | Germany | Thuringia | EPI_ISL_763004 | 2020-12-08 |
| **hCoV-19/Germany/TH-RKI-NP-0080/2020** | Germany | Thuringia | EPI_ISL_860299 | 2020-12-21 |
| **hCoV-19/Germany/SN-RKI-NP-0069/2020** | Germany | Saxony | EPI_ISL_763011 | 2020-12-17 |
| **hCoV-19/Germany/ST-MD202/2020** | Germany | Saxony-Anhalt | EPI_ISL_894161 | 2020-12-21 |
| **hCoV-19/Germany/ST-MD219/2020** | Germany | Saxony-Anhalt | EPI_ISL_894162 | 2020-12-18 |
| **hCoV-19/Germany/BB-RKI-NP-0056/2020** | Germany | Brandenburg | EPI_ISL_763057 | 2020-12-14 |
| **hCoV-19/Germany/BB-RKI-NP-0078/2020** | Germany | Brandenburg | EPI_ISL_860316 | 2020-12-21 |
| **hCoV-19/Germany/SN-RKI-Z-0042/2020** | Germany | Saxony | EPI_ISL_825165 | 2020-12-30 |
| **hCoV-19/Germany/ST-MD112/2020** | Germany | Saxony-Anhalt | EPI_ISL_803875 | 2020-12-07 |
| **hCoV-19/Germany/TH-RKI-NP-0062/2020** | Germany | Thuringia | EPI_ISL_763001 | 2020-12-01 |
| **hCoV-19/Germany/TH-RKI-NP-0018/2020** | Germany | Thuringia | EPI_ISL_763007 | 2020-12-01 |
| **hCoV-19/Germany/ST-MD195/2020** | Germany | Saxony-Anhalt | EPI_ISL_864578 | 2020-12-13 |
| **hCoV-19/Germany/ST-MD239/2020** | Germany | Saxony-Anhalt | EPI_ISL_884276 | 2020-12-28 |
| **hCoV-19/Germany/ST-MD212/2020** | Germany | Saxony-Anhalt | EPI_ISL_884261 | 2020-12-20 |
| **hCoV-19/Germany/ST-MD235/2020** | Germany | Saxony-Anhalt | EPI_ISL_884275 | 2020-12-28 |
| **hCoV-19/Germany/ST-MD226/2020** | Germany | Saxony-Anhalt | EPI_ISL_884262 | 2020-12-25 |
| **hCoV-19/Germany/ST-MD251/2020** | Germany | Saxony-Anhalt | EPI_ISL_884277 | 2020-12-31 |
| **hCoV-19/Germany/ST-MD228/2020** | Germany | Saxony-Anhalt | EPI_ISL_884263 | 2020-12-25 |
| **hCoV-19/Germany/ST-MD238/2020** | Germany | Saxony-Anhalt | EPI_ISL_884264 | 2020-12-28 |
| **hCoV-19/Germany/SN-RKI-Z-0044/2020** | Germany | Saxony | EPI_ISL_825171 | 2020-12-30 |
| **hCoV-19/Germany/ST-MD190/2020** | Germany | Saxony-Anhalt | EPI_ISL_864577 | 2020-12-01 |
| **hCoV-19/Germany/SN-RKI-NP-0024/2020** | Germany | Saxony | EPI_ISL_763019 | 2020-12-07 |
| **hCoV-19/Germany/ST-MD187/2020** | Germany | Saxony-Anhalt | EPI_ISL_864576 | 2020-12-14 |
| **hCoV-19/Germany/BB-RKI-NP-0055/2020** | Germany | Brandenburg | EPI_ISL_763058 | 2020-12-14 |
| **hCoV-19/Germany/SN-ChVir21664/2020** | Germany | Saxony | EPI_ISL_856683 | 2020-12-21 |
| **hCoV-19/Germany/SN-RKI-Z-0036/2020** | Germany | Saxony | EPI_ISL_825162 | 2020-12-23 |
| **hCoV-19/Germany/SN-RKI-Z-0035/2020** | Germany | Saxony | EPI_ISL_825161 | 2020-12-23 |
| **hCoV-19/Germany/SN-RKI-Z-0034/2020** | Germany | Saxony | EPI_ISL_825160 | 2020-12-20 |
| **hCoV-19/Germany/SN-RKI-Z-0032/2020** | Germany | Saxony | EPI_ISL_825159 | 2020-12-21 |
| **hCoV-19/Germany/SN-RKI-Z-0037/2020** | Germany | Saxony | EPI_ISL_825168 | 2020-12-23 |
| **hCoV-19/Germany/SN-RKI-Z-0041/2020** | Germany | Saxony | EPI_ISL_825169 | 2020-12-30 |
| **hCoV-19/Germany/SN-RKI-Z-0045/2020** | Germany | Saxony | EPI_ISL_825172 | 2020-12-30 |
| **hCoV-19/Germany/TH-hpi-p1690/2020** | Germany | Thuringia | EPI_ISL_776559 | 2020-03-18 |
| **hCoV-19/Germany/BE-ChVir-W1705-4345/2020** | Germany | Berlin | EPI_ISL_753750 | 2020-08-20 |
| **hCoV-19/Germany/BE-ChVir9004/2020** | Germany | Berlin | EPI_ISL_729601 | 2020-09-22 |
| **hCoV-19/Germany/TH-IIMK-CaSe-6/2020** | Germany | Thuringia | EPI_ISL_728296 | 2020-05-19 |
| **hCoV-19/Germany/BE-ChVir-D1996-4483/2020** | Germany | Berlin | EPI_ISL_753888 | 2020-10-13 |
| **hCoV-19/Germany/ST-MD118/2020** | Germany | Saxony-Anhalt | EPI_ISL_864544 | 2020-12-06 |
| **hCoV-19/Germany/ST-MD225/2020** | Germany | Saxony-Anhalt | EPI_ISL_894167 | 2020-12-25 |
| **hCoV-19/Germany/BE-ChVir-W2121-4431/2020** | Germany | Berlin | EPI_ISL_754186 | 2020-10-15 |
| **hCoV-19/Germany/BE-ChVir8998/2020** | Germany | Berlin | EPI_ISL_729370 | 2020-09-23 |
| **hCoV-19/Germany/BE-ChVir8996/2020** | Germany | Berlin | EPI_ISL_729408 | 2020-09-21 |
| **hCoV-19/Germany/BE-ChVir-D1981-5990/2020** | Germany | Berlin | EPI_ISL_754187 | 2020-09-24 |
| **hCoV-19/Germany/BE-ChVir-D1947-5915/2020** | Germany | Berlin | EPI_ISL_754189 | 2020-10-04 |
| **hCoV-19/Germany/BE-ChVir-D1971-7446/2020** | Germany | Berlin | EPI_ISL_754184 | 2020-10-04 |
| **hCoV-19/Germany/BE-ChVir-D1917-8291/2020** | Germany | Berlin | EPI_ISL_754188 | 2020-10-06 |
| **hCoV-19/Germany/BE-ChVir-W2120-5976/2020** | Germany | Berlin | EPI_ISL_754190 | 2020-10-15 |
| **hCoV-19/Germany/SN-RKI-NP-0034/2020** | Germany | Saxony | EPI_ISL_763015 | 2020-12-08 |
| **hCoV-19/Germany/BE-ChVir-W1302-8173/2020** | Germany | Berlin | EPI_ISL_753805 | 2020-04-29 |
| **hCoV-19/Germany/BE-ChVir-W1304-8152/2020** | Germany | Berlin | EPI_ISL_753806 | 2020-05-03 |
| **hCoV-19/Germany/BE-ChVir-W1532-5774/2020** | Germany | Berlin | EPI_ISL_753764 | 2020-04-05 |
| **hCoV-19/Germany/BE-RKI-Z-0015/2020** | Germany | Berlin | EPI_ISL_483150 | 2020-04-28 |
| **hCoV-19/Germany/BE-ChVir-W1447-8299/2020** | Germany | Berlin | EPI_ISL_753808 | 2020-06-23 |
| **hCoV-19/Germany/BE-ChVir-W1428-8266/2020** | Germany | Berlin | EPI_ISL_753807 | 2020-06-28 |
| **hCoV-19/Germany/BE-ChVir-W1648-8286/2020** | Germany | Berlin | EPI_ISL_753809 | 2020-07-01 |
| **hCoV-19/Germany/BE-ChVir-W1546-8284/2020** | Germany | Berlin | EPI_ISL_753736 | 2020-06-12 |
| **hCoV-19/Germany/BE-ChVir7621/2020** | Germany | Berlin | EPI_ISL_729555 | 2020-06-04 |
| **hCoV-19/Germany/BE-ChVir-W1440-8273/2020** | Germany | Berlin | EPI_ISL_753968 | 2020-06-18 |
| **hCoV-19/Germany/BE-ChVir7623/2020** | Germany | Berlin | EPI_ISL_729557 | 2020-06-05 |
| **hCoV-19/Germany/BE-ChVir-W1550-4329/2020** | Germany | Berlin | EPI_ISL_753755 | 2020-06-17 |
| **hCoV-19/Germany/BE-ChVir-W1547-4375/2020** | Germany | Berlin | EPI_ISL_753739 | 2020-06-11 |
| **hCoV-19/Germany/BE-ChVir-W1398-4386/2020** | Germany | Berlin | EPI_ISL_753826 | 2020-06-08 |
| **hCoV-19/Germany/BE-ChVir-D1948-8295/2020** | Germany | Berlin | EPI_ISL_753881 | 2020-10-05 |
| **hCoV-19/Germany/BE-ChVir-D1952-5748/2020** | Germany | Berlin | EPI_ISL_753882 | 2020-10-09 |
| **hCoV-19/Germany/BE-ChVir-W2117-4075/2020** | Germany | Berlin | EPI_ISL_754017 | 2020-10-12 |
| **hCoV-19/Germany/BE-ChVir-D1940-8248/2020** | Germany | Berlin | EPI_ISL_753876 | 2020-09-26 |
| **hCoV-19/Germany/BE-ChVir-D2000-8271/2020** | Germany | Berlin | EPI_ISL_753892 | 2020-10-15 |
| **hCoV-19/Germany/BE-ChVir-D1942-4496/2020** | Germany | Berlin | EPI_ISL_753878 | 2020-10-02 |
| **hCoV-19/Germany/BE-ChVir-D2033-5902/2020** | Germany | Berlin | EPI_ISL_753911 | 2020-10-06 |
| **hCoV-19/Germany/BB-ChVir-W2128-8294/2020** | Germany | Brandenburg | EPI_ISL_753859 | 2020-10-09 |
| **hCoV-19/Germany/BE-ChVir-D1953-7492/2020** | Germany | Berlin | EPI_ISL_753883 | 2020-10-10 |
| **hCoV-19/Germany/BE-ChVir-D1997-8209/2020** | Germany | Berlin | EPI_ISL_753889 | 2020-10-05 |
| **hCoV-19/Germany/BB-ChVir-W1067-7224/2020** | Germany | Brandenburg | EPI_ISL_753848 | 2020-04-01 |
| **hCoV-19/Germany/BE-RKI-N-0025/2020** | Germany | Berlin | EPI_ISL_481264 | 2020-03-27 |
| **hCoV-19/Germany/BB-ChVir-D933-5624/2020** | Germany | Brandenburg | EPI_ISL_753766 | 2020-03-14 |
| **hCoV-19/Germany/BE-ChVir-W1148-8141/2020** | Germany | Berlin | EPI_ISL_753942 | 2020-04-09 |
| **hCoV-19/Germany/BE-RKI-Z-0008/2020** | Germany | Berlin | EPI_ISL_483143 | 2020-04-21 |
| **hCoV-19/Germany/BE-RKI-Z-0009/2020** | Germany | Berlin | EPI_ISL_483144 | 2020-04-21 |
| **hCoV-19/Germany/BE-ChVir-W1542-8209/2020** | Germany | Berlin | EPI_ISL_753713 | 2020-06-23 |
| **hCoV-19/Germany/BE-ChVir-W1541-5685/2020** | Germany | Berlin | EPI_ISL_753748 | 2020-06-17 |
| **hCoV-19/Germany/BE-ChVir-W1416-4346/2020** | Germany | Berlin | EPI_ISL_753958 | 2020-06-22 |
| **hCoV-19/Germany/BE-ChVir-W1652-8241/2020** | Germany | Berlin | EPI_ISL_753983 | 2020-07-20 |
| **hCoV-19/Germany/BE-ChVir-W1077-8177/2020** | Germany | Berlin | EPI_ISL_753811 | 2020-04-04 |
| **hCoV-19/Germany/BE-ChVir-W1084-8118/2020** | Germany | Berlin | EPI_ISL_753935 | 2020-04-06 |
| **hCoV-19/Germany/BE-ChVir-W1142-8102/2020** | Germany | Berlin | EPI_ISL_753721 | 2020-04-07 |
| **hCoV-19/Germany/BE-ChVir-W1319-2159/2020** | Germany | Berlin | EPI_ISL_753823 | 2020-05-04 |
| **hCoV-19/Germany/BE-ChVir-W1396-4333/2020** | Germany | Berlin | EPI_ISL_753760 | 2020-05-11 |
| **hCoV-19/Germany/BE-ChVir-W1390-8235/2020** | Germany | Berlin | EPI_ISL_753723 | 2020-06-02 |
| **hCoV-19/Germany/BE-ChVir-W1536-4313/2020** | Germany | Berlin | EPI_ISL_753709 | 2020-05-17 |
| **hCoV-19/Germany/BE-ChVir-W1449-8226/2020** | Germany | Berlin | EPI_ISL_753971 | 2020-06-29 |
| **hCoV-19/Germany/BB-ChVir-W1545-4371/2020** | Germany | Brandenburg | EPI_ISL_753765 | 2020-06-19 |
| **hCoV-19/Germany/BE-ChVir7760/2020** | Germany | Berlin | EPI_ISL_729406 | 2020-06-19 |
| **hCoV-19/Germany/BE-ChVir-W1414-5697/2020** | Germany | Berlin | EPI_ISL_753956 | 2020-06-22 |
| **hCoV-19/Germany/BE-ChVir-W1427-8261/2020** | Germany | Berlin | EPI_ISL_753961 | 2020-06-25 |
| **hCoV-19/Germany/BE-ChVir-W1414-6707/2020** | Germany | Berlin | EPI_ISL_753957 | 2020-06-23 |
| **hCoV-19/Germany/BE-ChVir-W1551-4333/2020** | Germany | Berlin | EPI_ISL_753724 | 2020-06-20 |
| **hCoV-19/Germany/BE-ChVir-W1443-8219/2020** | Germany | Berlin | EPI_ISL_753969 | 2020-06-25 |
| **hCoV-19/Germany/BE-ChVir-W1445-6284/2020** | Germany | Berlin | EPI_ISL_753970 | 2020-06-29 |
| **hCoV-19/Germany/BE-ChVir-W1042-8106/2020** | Germany | Berlin | EPI_ISL_753923 | 2020-03-26 |
| **hCoV-19/Germany/SN-DcGC-L56891/2020** | Germany | Saxony | EPI_ISL_886157 | 2020-04-03 |
| **hCoV-19/Germany/SN-DcGC-L62649/2020** | Germany | Saxony | EPI_ISL_887108 | 2020-04-07 |
| **hCoV-19/Germany/BB-ChVir-D934-5623/2020** | Germany | Brandenburg | EPI_ISL_753813 | 2020-03-14 |
| **hCoV-19/Germany/BB-ChVir-D932-5620/2020** | Germany | Brandenburg | EPI_ISL_753770 | 2020-03-14 |
| **hCoV-19/Germany/BE-ChVir-W1064-4324/2020** | Germany | Berlin | EPI_ISL_753929 | 2020-04-01 |
| **hCoV-19/Germany/BE-ChVir-W1136-4353/2020** | Germany | Berlin | EPI_ISL_753938 | 2020-04-05 |
| **hCoV-19/Germany/BB-ChVir-W1073-8108/2020** | Germany | Brandenburg | EPI_ISL_753849 | 2020-04-03 |
| **hCoV-19/Germany/BB-ChVir3211/2020** | Germany | Brandenburg | EPI_ISL_729350 | 2020-03-23 |
| **hCoV-19/Germany/BB-ChVir-W1132-8159/2020** | Germany | Brandenburg | EPI_ISL_753718 | 2020-04-07 |
| **hCoV-19/Germany/BE-ChVir-W1055-8157/2020** | Germany | Berlin | EPI_ISL_753927 | 2020-03-30 |
| **hCoV-19/Germany/BE-ChVir-W1694-8256/2020** | Germany | Berlin | EPI_ISL_753989 | 2020-07-29 |
| **hCoV-19/Germany/BE-ChVir-W1698-8200/2020** | Germany | Berlin | EPI_ISL_753993 | 2020-07-30 |
| **hCoV-19/Germany/BE-ChVir-W1554-5041/2020** | Germany | Berlin | EPI_ISL_753705 | 2020-06-21 |
| **hCoV-19/Germany/BE-RKI-Z-0024/2020** | Germany | Berlin | EPI_ISL_483157 | 2020-04-28 |
| **hCoV-19/Germany/BE-ChVir-D1913-7445/2020** | Germany | Berlin | EPI_ISL_753861 | 2020-10-03 |
| **hCoV-19/Germany/BE-ChVir-D697-8137/2020** | Germany | Berlin | EPI_ISL_754055 | 2020-03-05 |
| **hCoV-19/Germany/BE-ChVir1919/2020** | Germany | Berlin | EPI_ISL_729487 | 2020-03-05 |
| **hCoV-19/Germany/BE-ChVir1716/2020** | Germany | Berlin | EPI_ISL_729481 | 2020-03-04 |
| **hCoV-19/Germany/BE-ChVir-D679-14/2020** | Germany | Berlin | EPI_ISL_516642 | 2020-03-04 |
| **hCoV-19/Germany/BE-ChVir-D672-4/2020** | Germany | Berlin | EPI_ISL_516632 | 2020-03-07 |
| **hCoV-19/Germany/BE-ChVir-D712-1/2020** | Germany | Berlin | EPI_ISL_516629 | 2020-03-07 |
| **hCoV-19/Germany/BE-ChVir-D718-3/2020** | Germany | Berlin | EPI_ISL_516631 | 2020-03-07 |
| **hCoV-19/Germany/BE-ChVir-D667-7/2020** | Germany | Berlin | EPI_ISL_516635 | 2020-03-07 |
| **hCoV-19/Germany/BE-ChVir-D666-2/2020** | Germany | Berlin | EPI_ISL_516630 | 2020-03-07 |
| **hCoV-19/Germany/BE-ChVir-D671-8/2020** | Germany | Berlin | EPI_ISL_516636 | 2020-03-07 |
| **hCoV-19/Germany/BE-ChVir-D710-10/2020** | Germany | Berlin | EPI_ISL_516638 | 2020-03-07 |
| **hCoV-19/Germany/BE-ChVir-D717-D761-12/2020** | Germany | Berlin | EPI_ISL_516640 | 2020-03-07 |
| **hCoV-19/Germany/BE-ChVir1685/2020** | Germany | Berlin | EPI_ISL_729479 | 2020-03-02 |
| **hCoV-19/Germany/BE-ChVir-D929-15/2020** | Germany | Berlin | EPI_ISL_516643 | 2020-03-05 |
| **hCoV-19/Germany/BE-ChVir1962/2020** | Germany | Berlin | EPI_ISL_729490 | 2020-03-07 |
| **hCoV-19/Germany/BE-ChVir-D670-9/2020** | Germany | Berlin | EPI_ISL_516637 | 2020-03-07 |
| **hCoV-19/Germany/BE-ChVir-W1078-7878/2020** | Germany | Berlin | EPI_ISL_753932 | 2020-04-04 |
| **hCoV-19/Germany/BE-ChVir-D793-8179/2020** | Germany | Berlin | EPI_ISL_753920 | 2020-03-13 |
| **hCoV-19/Germany/BE-ChVir-D903-4341/2020** | Germany | Berlin | EPI_ISL_753921 | 2020-03-15 |
| **hCoV-19/Germany/BE-ChVir-D909-4341/2020** | Germany | Berlin | EPI_ISL_753922 | 2020-03-16 |
| **hCoV-19/Germany/BE-ChVir-W1303-8106/2020** | Germany | Berlin | EPI_ISL_753952 | 2020-04-30 |
| **hCoV-19/Germany/BE-ChVir-W1531-4306/2020** | Germany | Berlin | EPI_ISL_753738 | 2020-04-28 |
| **hCoV-19/Germany/BE-ChVir-W1309-8155/2020** | Germany | Berlin | EPI_ISL_753953 | 2020-05-08 |
| **hCoV-19/Germany/BE-ChVir-W1299-4302/2020** | Germany | Berlin | EPI_ISL_753951 | 2020-04-28 |
| **hCoV-19/Germany/BB-ChVir-W1066-7201/2020** | Germany | Brandenburg | EPI_ISL_753847 | 2020-04-01 |
| **hCoV-19/Germany/BB-ChVir-W1052-7250/2020** | Germany | Brandenburg | EPI_ISL_753844 | 2020-03-28 |
| **hCoV-19/Germany/BE-ChVir-W1045-5646/2020** | Germany | Berlin | EPI_ISL_753925 | 2020-03-26 |
| **hCoV-19/Germany/BE-ChVir-3185-a-7481/2020** | Germany | Berlin | EPI_ISL_806535 | 2020-04-14 |
| **hCoV-19/Germany/BE-RKI-Z-0010/2020** | Germany | Berlin | EPI_ISL_483145 | 2020-04-25 |
| **hCoV-19/Germany/BE-ChVir-W1315-5629/2020** | Germany | Berlin | EPI_ISL_753753 | 2020-04-16 |
| **hCoV-19/Germany/BE-ChVir-W1297-7420/2020** | Germany | Berlin | EPI_ISL_753950 | 2020-04-27 |
| **hCoV-19/Germany/BE-RKI-Z-0011/2020** | Germany | Berlin | EPI_ISL_483146 | 2020-04-25 |
| **hCoV-19/Germany/BE-RKI-Z-0012/2020** | Germany | Berlin | EPI_ISL_483147 | 2020-04-25 |
| **hCoV-19/Germany/BE-ChVir-3170-a-4370/2020** | Germany | Berlin | EPI_ISL_806528 | 2020-03-26 |
| **hCoV-19/Germany/BE-ChVir-D749-5790/2020** | Germany | Berlin | EPI_ISL_753917 | 2020-03-29 |
| **hCoV-19/Germany/BE-ChVir3106/2020** | Germany | Berlin | EPI_ISL_729533 | 2020-03-29 |
| **hCoV-19/Germany/BB-ChVir-3187-a-8159/2020** | Germany | Brandenburg | EPI_ISL_806537 | 2020-04-14 |
| **hCoV-19/Germany/BE-ChVir-W1321-4373/2020** | Germany | Berlin | EPI_ISL_753772 | 2020-05-04 |
| **hCoV-19/Germany/BE-ChVir-D750-8181/2020** | Germany | Berlin | EPI_ISL_754056 | 2020-03-15 |
| **hCoV-19/Germany/BE-ChVir1900/2020** | Germany | Berlin | EPI_ISL_729483 | 2020-03-17 |
| **hCoV-19/Germany/BE-RKI-Z-0017/2020** | Germany | Berlin | EPI_ISL_483151 | 2020-04-28 |
| **hCoV-19/Germany/BE-ChVir3083/2020** | Germany | Berlin | EPI_ISL_729529 | 2020-03-24 |
| **hCoV-19/Germany/BE-ChVir-W1063-8162/2020** | Germany | Berlin | EPI_ISL_753729 | 2020-04-05 |
| **hCoV-19/Germany/SN-DcGC-L62670/2020** | Germany | Saxony | EPI_ISL_887111 | 2020-04-21 |
| **hCoV-19/Germany/TH-IIMK-CaSe-7/2020** | Germany | Thuringia | EPI_ISL_728298 | 2020-05-19 |
| **hCoV-19/Germany/BE-ChVir-W1301-8113/2020** | Germany | Berlin | EPI_ISL_753800 | 2020-04-30 |
| **hCoV-19/Germany/BE-ChVir-3190-a-4383/2020** | Germany | Berlin | EPI_ISL_806539 | 2020-04-02 |
| **hCoV-19/Germany/BE-ChVir-3174-a-7294/2020** | Germany | Berlin | EPI_ISL_806530 | 2020-04-02 |
| **hCoV-19/Germany/BE-ChVir-W1178-8106/2020** | Germany | Berlin | EPI_ISL_753945 | 2020-04-01 |
| **hCoV-19/Germany/BE-ChVir-W1137-5740/2020** | Germany | Berlin | EPI_ISL_753939 | 2020-04-09 |
| **hCoV-19/Germany/BE-ChVir-W1133-5742/2020** | Germany | Berlin | EPI_ISL_753936 | 2020-03-31 |
| **hCoV-19/Germany/BB-ChVir-W1061-7256/2020** | Germany | Brandenburg | EPI_ISL_753845 | 2020-03-30 |
| **hCoV-19/Germany/BE-ChVir-D665-6/2020** | Germany | Berlin | EPI_ISL_516634 | 2020-03-06 |
| **hCoV-19/Germany/BE-ChVir-D658-5/2020** | Germany | Berlin | EPI_ISL_516633 | 2020-03-08 |
| **hCoV-19/Germany/BE-ChVir1968/2020** | Germany | Berlin | EPI_ISL_729492 | 2020-03-08 |
| **hCoV-19/Germany/BE-ChVir-W1191-13/2020** | Germany | Berlin | EPI_ISL_516641 | 2020-03-08 |
| **hCoV-19/Germany/BB-ChVir-W1129-7275/2020** | Germany | Brandenburg | EPI_ISL_753850 | 2020-03-31 |
| **hCoV-19/Germany/BB-ChVir-W1135-7242/2020** | Germany | Brandenburg | EPI_ISL_753851 | 2020-03-31 |
| **hCoV-19/Germany/BE-ChVir3100/2020** | Germany | Berlin | EPI_ISL_729531 | 2020-03-24 |
| **hCoV-19/Germany/BE-ChVir-W1149-8118/2020** | Germany | Berlin | EPI_ISL_753943 | 2020-04-09 |
| **hCoV-19/Germany/BE-ChVir-W1043-8183/2020** | Germany | Berlin | EPI_ISL_753924 | 2020-03-26 |
| **hCoV-19/Germany/TH-IIMK-CaSe-23/2020** | Germany | Thuringia | EPI_ISL_763088 | 2020-03-24 |
| **hCoV-19/Germany/TH-IIMK-CaSe-17/2020** | Germany | Thuringia | EPI_ISL_728338 | 2020-12-06 |
| **hCoV-19/Germany/TH-IIMK-CaSe-16/2020** | Germany | Thuringia | EPI_ISL_728337 | 2020-12-06 |
| **hCoV-19/Germany/TH-IIMK-CaSe-19/2020** | Germany | Thuringia | EPI_ISL_728345 | 2020-12-06 |
| **hCoV-19/Germany/BE-ChVir-D1982-5039/2020** | Germany | Berlin | EPI_ISL_753743 | 2020-09-25 |
| **hCoV-19/Germany/BE-ChVir-W1716-6716/2020** | Germany | Berlin | EPI_ISL_754006 | 2020-08-16 |
| **hCoV-19/Germany/SN-DcGC-L62674/2020** | Germany | Saxony | EPI_ISL_887113 | 2020-10-04 |
| **hCoV-19/Germany/BE-ChVir8992/2020** | Germany | Berlin | EPI_ISL_729377 | 2020-09-21 |
| **hCoV-19/Germany/SN-RKI-NP-0028/2020** | Germany | Saxony | EPI_ISL_763018 | 2020-12-08 |
| **hCoV-19/Germany/SN-RKI-NP-0070/2020** | Germany | Saxony | EPI_ISL_763010 | 2020-12-17 |
| **hCoV-19/Germany/SN-RKI-NP-0042/2020** | Germany | Saxony | EPI_ISL_763014 | 2020-12-09 |
| **hCoV-19/Germany/TH-IIMK-CaSe-13/2020** | Germany | Thuringia | EPI_ISL_728331 | 2020-12-01 |
| **hCoV-19/Germany/TH-IIMK-CaSe-14/2020** | Germany | Thuringia | EPI_ISL_728333 | 2020-12-01 |
| **hCoV-19/Germany/SN-RKI-NP-0065/2020** | Germany | Saxony | EPI_ISL_763012 | 2020-12-15 |
| **hCoV-19/Germany/SN-RKI-NP-0063/2020** | Germany | Saxony | EPI_ISL_763013 | 2020-12-15 |
| **hCoV-19/Germany/TH-IIMK-CaSe-5/2020** | Germany | Thuringia | EPI_ISL_728294 | 2020-05-19 |
| **hCoV-19/Germany/ST-MD216/2020** | Germany | Saxony-Anhalt | EPI_ISL_894166 | 2020-12-21 |
| **hCoV-19/Germany/BE-ChVir-W1731-2742/2020** | Germany | Berlin | EPI_ISL_754016 | 2020-08-07 |
| **hCoV-19/Germany/TH-ChVir21797/2020** | Germany | Thuringia | EPI_ISL_849842 | 2020-12-26 |
| **hCoV-19/Germany/TH-RKI-NP-0022/2020** | Germany | Thuringia | EPI_ISL_763005 | 2020-12-07 |
| **hCoV-19/Germany/TH-RKI-I-017493/2020** | Germany | Thuringia | EPI_ISL_1147655 | 2020-12-10 |
| **hCoV-19/Germany/TH-RKI-I-017494/2020** | Germany | Thuringia | EPI_ISL_1147656 | 2020-12-10 |
| **hCoV-19/Germany/BE-ChVir-D2004-8247/2020** | Germany | Berlin | EPI_ISL_753894 | 2020-10-19 |
| **hCoV-19/Germany/ST-MD222/2020** | Germany | Saxony-Anhalt | EPI_ISL_884284 | 2020-12-25 |
| **hCoV-19/Germany/BE-ChVir9036/2020** | Germany | Berlin | EPI_ISL_729410 | 2020-09-20 |
| **hCoV-19/Germany/BE-ChVir-D1957-4437/2020** | Germany | Berlin | EPI_ISL_753885 | 2020-09-26 |
| **hCoV-19/Germany/BE-ChVir-D1980-8244/2020** | Germany | Berlin | EPI_ISL_753751 | 2020-09-28 |
| **hCoV-19/Germany/BE-ChVir-D2046-8225/2020** | Germany | Berlin | EPI_ISL_753707 | 2020-10-12 |
| **hCoV-19/Germany/BE-ChVir-D2011-5016/2020** | Germany | Berlin | EPI_ISL_753899 | 2020-10-15 |
| **hCoV-19/Germany/BE-ChVir9010/2020** | Germany | Berlin | EPI_ISL_729398 | 2020-09-22 |
| **hCoV-19/Germany/SN-RKI-NP-0076/2020** | Germany | Saxony | EPI_ISL_763009 | 2020-12-21 |
| **hCoV-19/Germany/BE-ChVir-D1916-8268/2020** | Germany | Berlin | EPI_ISL_753863 | 2020-10-06 |
| **hCoV-19/Germany/BE-ChVir-D2006-8256/2020** | Germany | Berlin | EPI_ISL_753895 | 2020-10-15 |
| **hCoV-19/Germany/BE-ChVir-D1956-8255/2020** | Germany | Berlin | EPI_ISL_753884 | 2020-09-25 |
| **hCoV-19/Germany/BE-ChVir-D1976-8223/2020** | Germany | Berlin | EPI_ISL_753717 | 2020-10-08 |
| **hCoV-19/Germany/BE-ChVir-D2034-8234/2020** | Germany | Berlin | EPI_ISL_753912 | 2020-10-13 |
| **hCoV-19/Germany/BE-ChVir-W2124-5942/2020** | Germany | Berlin | EPI_ISL_754020 | 2020-10-18 |
| **hCoV-19/Germany/BE-ChVir-D2026-8257/2020** | Germany | Berlin | EPI_ISL_753909 | 2020-10-14 |
| **hCoV-19/Germany/BE-ChVir-D2016-5933/2020** | Germany | Berlin | EPI_ISL_753901 | 2020-10-18 |
| **hCoV-19/Germany/BE-ChVir-D1977-5942/2020** | Germany | Berlin | EPI_ISL_753816 | 2020-10-08 |
| **hCoV-19/Germany/BE-ChVir-D2025-5941/2020** | Germany | Berlin | EPI_ISL_753908 | 2020-10-08 |
| **hCoV-19/Germany/BE-ChVir-D2023-4491/2020** | Germany | Berlin | EPI_ISL_753906 | 2020-10-11 |
| **hCoV-19/Germany/BE-ChVir9039/2020** | Germany | Berlin | EPI_ISL_729357 | 2020-09-21 |
| **hCoV-19/Germany/TH-IIMK-CaSe-15/2020** | Germany | Thuringia | EPI_ISL_728335 | 2020-12-03 |
| **hCoV-19/Germany/TH-RKI-NP-0072/2020** | Germany | Thuringia | EPI_ISL_763000 | 2020-12-16 |
| **hCoV-19/Germany/TH-RKI-NP-0081/2020** | Germany | Thuringia | EPI_ISL_860300 | 2020-12-22 |
| **hCoV-19/Germany/BE-ChVir21815/2020** | Germany | Berlin | EPI_ISL_849858 | 2020-12-26 |
| **hCoV-19/Germany/ST-MD258/2020** | Germany | Saxony-Anhalt | EPI_ISL_884288 | 2020-12-28 |
| **hCoV-19/Germany/ST-MD257/2020** | Germany | Saxony-Anhalt | EPI_ISL_894169 | 2020-12-28 |
| **hCoV-19/Germany/SN-DcGC-L62664/2020** | Germany | Saxony | EPI_ISL_887109 | 2020-09-10 |
| **hCoV-19/Germany/BE-ChVir8986/2020** | Germany | Berlin | EPI_ISL_729348 | 2020-09-21 |
| **hCoV-19/Germany/TH-IIMK-CaSe-29/2020** | Germany | Thuringia | EPI_ISL_768633 | 2020-12-08 |
| **hCoV-19/Germany/BE-ChVir-D2003-5974/2020** | Germany | Berlin | EPI_ISL_753893 | 2020-10-15 |
| **hCoV-19/Germany/BE-ChVir8988/2020** | Germany | Berlin | EPI_ISL_729599 | 2020-09-20 |
| **hCoV-19/Germany/BB-RKI-NP-0067/2020** | Germany | Brandenburg | EPI_ISL_763054 | 2020-12-17 |
| **hCoV-19/Germany/BE-RKI-N-0158/2020** | Germany | Berlin | EPI_ISL_1048332 | 2020-10-08 |
| **hCoV-19/Germany/BE-ChVir-W2122-5977/2020** | Germany | Berlin | EPI_ISL_754018 | 2020-10-15 |
| **hCoV-19/Germany/BE-ChVir8983/2020** | Germany | Berlin | EPI_ISL_729366 | 2020-09-21 |
| **hCoV-19/Germany/BE-ChVir9030/2020** | Germany | Berlin | EPI_ISL_729606 | 2020-09-21 |
| **hCoV-19/Germany/BE-ChVir9009/2020** | Germany | Berlin | EPI_ISL_729603 | 2020-09-22 |
| **hCoV-19/Germany/BE-ChVir-D1936-8209/2020** | Germany | Berlin | EPI_ISL_753785 | 2020-09-24 |
| **hCoV-19/Germany/BE-ChVir-D1938-8233/2020** | Germany | Berlin | EPI_ISL_753786 | 2020-09-28 |
| **hCoV-19/Germany/BE-ChVir-D2014-8229/2020** | Germany | Berlin | EPI_ISL_753792 | 2020-10-14 |
| **hCoV-19/Germany/BE-ChVir-D1946-8235/2020** | Germany | Berlin | EPI_ISL_753788 | 2020-10-01 |
| **hCoV-19/Germany/BE-ChVir-D2001-8219/2020** | Germany | Berlin | EPI_ISL_753790 | 2020-10-19 |
| **hCoV-19/Germany/BE-ChVir-D1934-8278/2020** | Germany | Berlin | EPI_ISL_753784 | 2020-10-11 |
| **hCoV-19/Germany/BB-RKI-NP-0038/2020** | Germany | Brandenburg | EPI_ISL_763059 | 2020-12-10 |
| **hCoV-19/Germany/BE-RKI-N-0163/2020** | Germany | Berlin | EPI_ISL_1048307 | 2020-10-26 |
| **hCoV-19/Germany/ST-MD252/2020** | Germany | Saxony-Anhalt | EPI_ISL_884274 | 2020-12-31 |
| **hCoV-19/Germany/BE-ChVir-D1933-8292/2020** | Germany | Berlin | EPI_ISL_753783 | 2020-10-08 |
| **hCoV-19/Germany/BE-ChVir-D1929-7417/2020** | Germany | Berlin | EPI_ISL_753781 | 2020-10-09 |
| **hCoV-19/Germany/SN-RKI-Z-0033/2020** | Germany | Saxony | EPI_ISL_825166 | 2020-12-21 |
| **hCoV-19/Germany/BE-ChVir-D2032-8244/2020** | Germany | Berlin | EPI_ISL_753794 | 2020-10-12 |
| **hCoV-19/Germany/TH-RKI-NP-0048/2020** | Germany | Thuringia | EPI_ISL_763003 | 2020-12-11 |
| **hCoV-19/Germany/ST-MD229/2020** | Germany | Saxony-Anhalt | EPI_ISL_884272 | 2020-12-27 |
| **hCoV-19/Germany/BE-ChVir-D1939-8206/2020** | Germany | Berlin | EPI_ISL_753787 | 2020-09-26 |
| **hCoV-19/Germany/BB-ChVir-D2047-5009/2020** | Germany | Brandenburg | EPI_ISL_753719 | 2020-10-15 |
| **hCoV-19/Germany/BE-ChVir-D1931-8262/2020** | Germany | Berlin | EPI_ISL_753782 | 2020-10-08 |
| **hCoV-19/Germany/BE-ChVir-D2029-8271/2020** | Germany | Berlin | EPI_ISL_753793 | 2020-10-15 |
| **hCoV-19/Germany/BE-ChVir-W2119-8203/2020** | Germany | Berlin | EPI_ISL_753711 | 2020-10-14 |
| **hCoV-19/Germany/BB-RKI-NP-0035/2020** | Germany | Brandenburg | EPI_ISL_763061 | 2020-12-09 |
| **hCoV-19/Germany/BE-ChVir-W2118-4404/2020** | Germany | Berlin | EPI_ISL_753795 | 2020-10-12 |
| **hCoV-19/Germany/BE-ChVir-D1955-8202/2020** | Germany | Berlin | EPI_ISL_753789 | 2020-10-11 |
| **hCoV-19/Germany/BE-ChVir-D2028-8264/2020** | Germany | Berlin | EPI_ISL_754191 | 2020-10-14 |
| **hCoV-19/Germany/BE-ChVir-D1954-5097/2020** | Germany | Berlin | EPI_ISL_753780 | 2020-10-11 |
| **hCoV-19/Germany/ST-MD206/2020** | Germany | Saxony-Anhalt | EPI_ISL_884266 | 2020-12-18 |
| **hCoV-19/Germany/BE-ChVir21646/2020** | Germany | Berlin | EPI_ISL_862145 | 2020-12-24 |
| **hCoV-19/Germany/BE-ChVir-D2009-8236/2020** | Germany | Berlin | EPI_ISL_753791 | 2020-10-15 |
| **hCoV-19/Germany/BE-ChVir-D1951-5060/2020** | Germany | Berlin | EPI_ISL_753779 | 2020-10-10 |
| **hCoV-19/Germany/TH-IIMK-CaSe-18/2020** | Germany | Thuringia | EPI_ISL_728341 | 2020-12-06 |
| **hCoV-19/Germany/ST-MD244/2020** | Germany | Saxony-Anhalt | EPI_ISL_884268 | 2020-12-29 |
| **hCoV-19/Germany/ST-MD249/2020** | Germany | Saxony-Anhalt | EPI_ISL_884287 | 2020-12-31 |
| **hCoV-19/Germany/ST-MD230/2020** | Germany | Saxony-Anhalt | EPI_ISL_884267 | 2020-12-26 |
| **hCoV-19/Germany/ST-MD240/2020** | Germany | Saxony-Anhalt | EPI_ISL_884273 | 2020-12-28 |
| **hCoV-19/Germany/ST-MD237/2020** | Germany | Saxony-Anhalt | EPI_ISL_884260 | 2020-12-28 |
| **hCoV-19/Germany/ST-MD234/2020** | Germany | Saxony-Anhalt | EPI_ISL_884271 | 2020-12-26 |
| **hCoV-19/Germany/TH-IIMK-CaSe-10/2020** | Germany | Thuringia | EPI_ISL_728321 | 2020-05-24 |
| **hCoV-19/Germany/SN-DcGC-L56902/2020** | Germany | Saxony | EPI_ISL_887107 | 2020-04-06 |
| **hCoV-19/Germany/BE-ChVir-W1291-8183/2020** | Germany | Berlin | EPI_ISL_753949 | 2020-04-21 |
| **hCoV-19/Germany/BE-ChVir-D792-7028/2020** | Germany | Berlin | EPI_ISL_753919 | 2020-03-14 |
| **hCoV-19/Germany/BE-ChVir1970/2020** | Germany | Berlin | EPI_ISL_729493 | 2020-03-08 |
| **hCoV-19/Germany/BE-ChVir-W1706-8239/2020** | Germany | Berlin | EPI_ISL_753997 | 2020-08-15 |
| **hCoV-19/Germany/BE-ChVir-3184-a-4310/2020** | Germany | Berlin | EPI_ISL_806534 | 2020-04-13 |
| **hCoV-19/Germany/BB-RKI-N-0157/2020** | Germany | Brandenburg | EPI_ISL_1048333 | 2020-10-06 |
| **hCoV-19/Germany/BE-RKI-Z-0031/2020** | Germany | Berlin | EPI_ISL_803894 | 2020-12-28 |
| **hCoV-19/Germany/BE-ChVir-W1134-8182/2020** | Germany | Berlin | EPI_ISL_753937 | 2020-04-01 |
| **hCoV-19/Germany/BE-ChVir-3179-a-8183/2020** | Germany | Berlin | EPI_ISL_806533 | 2020-04-05 |
| **hCoV-19/Germany/BE-ChVir-3193-8104/2020** | Germany | Berlin | EPI_ISL_806527 | 2020-04-02 |
| **hCoV-19/Germany/BB-ChVir-W1144-7244/2020** | Germany | Brandenburg | EPI_ISL_753852 | 2020-04-08 |
| **hCoV-19/Germany/BE-ChVir-D1928-7459/2020** | Germany | Berlin | EPI_ISL_753872 | 2020-10-09 |
| **hCoV-19/Germany/BE-ChVir-W1340-8291/2020** | Germany | Berlin | EPI_ISL_753955 | 2020-05-12 |
| **hCoV-19/Germany/BE-ChVir-W1722-6784/2020** | Germany | Berlin | EPI_ISL_754011 | 2020-08-16 |
| **hCoV-19/Germany/BE-ChVir-W1693-4382/2020** | Germany | Berlin | EPI_ISL_753988 | 2020-08-04 |
| **hCoV-19/Germany/BE-ChVir-W1712-4302/2020** | Germany | Berlin | EPI_ISL_754002 | 2020-08-07 |
| **hCoV-19/Germany/BE-ChVir-W1151-4300/2020** | Germany | Berlin | EPI_ISL_753944 | 2020-04-12 |
| **hCoV-19/Germany/BE-ChVir-W1282-4384/2020** | Germany | Berlin | EPI_ISL_753947 | 2020-04-17 |
| **hCoV-19/Germany/BB-ChVir-W1076-4385/2020** | Germany | Brandenburg | EPI_ISL_753774 | 2020-04-04 |
| **hCoV-19/Germany/BE-ChVir-3192-a-4365/2020** | Germany | Berlin | EPI_ISL_806540 | 2020-04-02 |
| **hCoV-19/Germany/SN-Cento-35080433/2020** | Germany | Saxony | EPI_ISL_815312 | 2020-09-25 |
| **hCoV-19/Germany/BE-ChVir-W1697-8293/2020** | Germany | Berlin | EPI_ISL_753992 | 2020-07-13 |
| **hCoV-19/Germany/BE-ChVir-W2125-4401/2020** | Germany | Berlin | EPI_ISL_754021 | 2020-09-27 |
| **hCoV-19/Germany/BE-ChVir-D2012-8249/2020** | Germany | Berlin | EPI_ISL_753900 | 2020-10-15 |
| **hCoV-19/Germany/BE-ChVir-W1555-7451/2020** | Germany | Berlin | EPI_ISL_753757 | 2020-06-23 |
| **hCoV-19/Germany/TH-MMV-Bo-1/2020** | Germany | Thuringia | EPI_ISL_1118929 | 2020-07-17 |
| **hCoV-19/Germany/BE-ChVir-W1684-5684/2020** | Germany | Berlin | EPI_ISL_753778 | 2020-07-20 |
| **hCoV-19/Germany/BE-ChVir-W1718-6763/2020** | Germany | Berlin | EPI_ISL_753810 | 2020-08-19 |
| **hCoV-19/Germany/BE-ChVir-W1679-4309/2020** | Germany | Berlin | EPI_ISL_753768 | 2020-08-01 |
| **hCoV-19/Germany/BE-ChVir-W1713-6783/2020** | Germany | Berlin | EPI_ISL_754003 | 2020-08-05 |
| **hCoV-19/Germany/BE-ChVir-D1984-4423/2020** | Germany | Berlin | EPI_ISL_753820 | 2020-09-26 |
| **hCoV-19/Germany/BE-ChVir-D1959-4442/2020** | Germany | Berlin | EPI_ISL_753887 | 2020-09-24 |
| **hCoV-19/Germany/BE-ChVir-D1988-4466/2020** | Germany | Berlin | EPI_ISL_753821 | 2020-10-01 |
| **hCoV-19/Germany/BE-ChVir-D1970-8225/2020** | Germany | Berlin | EPI_ISL_753758 | 2020-10-03 |
| **hCoV-19/Germany/BE-ChVir8991/2020** | Germany | Berlin | EPI_ISL_729600 | 2020-09-21 |
| **hCoV-19/Germany/BE-ChVir-D1941-8263/2020** | Germany | Berlin | EPI_ISL_753877 | 2020-09-24 |
| **hCoV-19/Germany/TH-IIMK-CaSe-3/2020** | Germany | Thuringia | EPI_ISL_728288 | 2020-04-03 |
| **hCoV-19/Germany/TH-IIMK-CaSe-4/2020** | Germany | Thuringia | EPI_ISL_728293 | 2020-04-03 |
| **hCoV-19/Germany/BE-ChVir-D2021-4462/2020** | Germany | Berlin | EPI_ISL_753904 | 2020-10-10 |
| **hCoV-19/Germany/BE-ChVir-W1700-6762/2020** | Germany | Berlin | EPI_ISL_753994 | 2020-08-09 |
| **hCoV-19/Germany/BE-ChVir-D1944-8293/2020** | Germany | Berlin | EPI_ISL_753777 | 2020-10-01 |
| **hCoV-19/Germany/BE-ChVir-W1642-6757/2020** | Germany | Berlin | EPI_ISL_753977 | 2020-08-05 |
| **hCoV-19/Germany/TH-Cento-36547931/2020** | Germany | Thuringia | EPI_ISL_815321 | 2020-07-11 |
| **hCoV-19/Germany/BE-ChVir-W1644-6714/2020** | Germany | Berlin | EPI_ISL_753978 | 2020-08-05 |
| **hCoV-19/Germany/BE-ChVir-W1721-6777/2020** | Germany | Berlin | EPI_ISL_754010 | 2020-08-14 |
| **hCoV-19/Germany/BE-ChVir-W1720-6773/2020** | Germany | Berlin | EPI_ISL_754009 | 2020-08-14 |
| **hCoV-19/Germany/ST-ChVir-W1702-6718/2020** | Germany | Saxony-Anhalt | EPI_ISL_754054 | 2020-08-09 |
| **hCoV-19/Germany/BE-ChVir-W1691-8296/2020** | Germany | Berlin | EPI_ISL_753986 | 2020-07-31 |
| **hCoV-19/Germany/BE-ChVir-D1918-8214/2020** | Germany | Berlin | EPI_ISL_753864 | 2020-10-06 |
| **hCoV-19/Germany/BE-ChVir-D1932-8298/2020** | Germany | Berlin | EPI_ISL_753874 | 2020-10-08 |
| **hCoV-19/Germany/BE-ChVir-3182-a-8148/2020** | Germany | Berlin | EPI_ISL_806542 | 2020-04-13 |
| **hCoV-19/Germany/BE-ChVir-3177-a-4339/2020** | Germany | Berlin | EPI_ISL_806531 | 2020-04-02 |
| **hCoV-19/Germany/BE-ChVir-W1389-4345/2020** | Germany | Berlin | EPI_ISL_753825 | 2020-05-20 |
| **hCoV-19/Germany/BE-RKI-Z-0014/2020** | Germany | Berlin | EPI_ISL_483149 | 2020-04-27 |
| **hCoV-19/Germany/TH-IIMK-CaSe-9/2020** | Germany | Thuringia | EPI_ISL_728303 | 2020-05-24 |
| **hCoV-19/Germany/BE-ChVir-W1727-8260/2020** | Germany | Berlin | EPI_ISL_754014 | 2020-08-19 |
| **hCoV-19/Germany/BE-ChVir-W1552-7013/2020** | Germany | Berlin | EPI_ISL_753720 | 2020-06-17 |
| **hCoV-19/Germany/BB-ChVir-W1419-8228/2020** | Germany | Brandenburg | EPI_ISL_753854 | 2020-06-18 |
| **hCoV-19/Germany/BE-ChVir-W1433-8258/2020** | Germany | Berlin | EPI_ISL_753965 | 2020-06-17 |
| **hCoV-19/Germany/BE-ChVir-W1431-8290/2020** | Germany | Berlin | EPI_ISL_753963 | 2020-06-19 |
| **hCoV-19/Germany/BB-ChVir-W1413-8296/2020** | Germany | Brandenburg | EPI_ISL_753853 | 2020-06-19 |
| **hCoV-19/Germany/BE-ChVir-W1415-6227/2020** | Germany | Berlin | EPI_ISL_753706 | 2020-06-22 |
| **hCoV-19/Germany/BE-ChVir-W1432-4374/2020** | Germany | Berlin | EPI_ISL_753964 | 2020-06-26 |
| **hCoV-19/Germany/BE-ChVir-W1451-8252/2020** | Germany | Berlin | EPI_ISL_753972 | 2020-06-29 |
| **hCoV-19/Germany/ST-MD328/2020** | Germany | Saxony-Anhalt | EPI_ISL_960867 | 2020-12-25 |
| **hCoV-19/Germany/BE-ChVir-W1649-7416/2020** | Germany | Berlin | EPI_ISL_753981 | 2020-07-16 |
| **hCoV-19/Germany/BE-ChVir-W1436-4360/2020** | Germany | Berlin | EPI_ISL_753966 | 2020-06-16 |
| **hCoV-19/Germany/BE-ChVir-W1588-8297/2020** | Germany | Berlin | EPI_ISL_753726 | 2020-06-30 |
| **hCoV-19/Germany/BB-ChVir-W1442-8234/2020** | Germany | Brandenburg | EPI_ISL_753857 | 2020-06-19 |
| **hCoV-19/Germany/BE-ChVir-W1695-8202/2020** | Germany | Berlin | EPI_ISL_753990 | 2020-07-01 |
| **hCoV-19/Germany/BE-ChVir-W1556-6773/2020** | Germany | Berlin | EPI_ISL_753733 | 2020-06-23 |
| **hCoV-19/Germany/BE-ChVir-W1548-8216/2020** | Germany | Berlin | EPI_ISL_753744 | 2020-06-19 |
| **hCoV-19/Germany/BB-ChVir-W1423-8226/2020** | Germany | Brandenburg | EPI_ISL_753855 | 2020-06-19 |
| **hCoV-19/Germany/BE-ChVir-W1418-8203/2020** | Germany | Berlin | EPI_ISL_753960 | 2020-06-30 |
| **hCoV-19/Germany/BE-ChVir-W1429-8283/2020** | Germany | Berlin | EPI_ISL_753962 | 2020-06-27 |
| **hCoV-19/Germany/BB-ChVir-W1549-7455/2020** | Germany | Brandenburg | EPI_ISL_753771 | 2020-06-17 |
| **hCoV-19/Germany/BE-ChVir-W1696-8291/2020** | Germany | Berlin | EPI_ISL_753991 | 2020-07-23 |
| **hCoV-19/Germany/BE-ChVir-W1688-4369/2020** | Germany | Berlin | EPI_ISL_753984 | 2020-07-16 |
| **hCoV-19/Germany/BE-ChVir-W1544-8280/2020** | Germany | Berlin | EPI_ISL_753756 | 2020-06-22 |
| **hCoV-19/Germany/BB-ChVir-W1426-8229/2020** | Germany | Brandenburg | EPI_ISL_753856 | 2020-06-19 |
| **hCoV-19/Germany/BE-ChVir-W1651-8237/2020** | Germany | Berlin | EPI_ISL_753982 | 2020-07-20 |
| **hCoV-19/Germany/BE-RKI-Z-0019/2020** | Germany | Berlin | EPI_ISL_483153 | 2020-04-21 |
| **hCoV-19/Germany/BE-ChVir-W1690-8217/2020** | Germany | Berlin | EPI_ISL_753985 | 2020-07-17 |
| **hCoV-19/Germany/SN-DcGC-L62673/2020** | Germany | Saxony | EPI_ISL_887112 | 2020-10-03 |
| **hCoV-19/Germany/TH-IIMK-CaSe-8/2020** | Germany | Thuringia | EPI_ISL_728302 | 2020-05-19 |
| **hCoV-19/Germany/BE-ChVir-W1704-4391/2020** | Germany | Berlin | EPI_ISL_753996 | 2020-08-09 |
| **hCoV-19/Germany/SN-ChVir-W1155-4392/2020** | Germany | Saxony | EPI_ISL_754050 | 2020-04-07 |
| **hCoV-19/Germany/SN-ChVir-W1276-4387/2020** | Germany | Saxony | EPI_ISL_754053 | 2020-04-15 |
| **hCoV-19/Germany/SN-ChVir-W1314-4321/2020** | Germany | Saxony | EPI_ISL_753798 | 2020-04-17 |
| **hCoV-19/Germany/SN-ChVir-W1313-4308/2020** | Germany | Saxony | EPI_ISL_753797 | 2020-04-16 |
| **hCoV-19/Germany/SN-ChVir-W1157-4330/2020** | Germany | Saxony | EPI_ISL_754051 | 2020-04-06 |
| **hCoV-19/Germany/SN-ChVir-W1312-4386/2020** | Germany | Saxony | EPI_ISL_753836 | 2020-04-15 |
| **hCoV-19/Germany/SN-ChVir-W1153-4377/2020** | Germany | Saxony | EPI_ISL_754049 | 2020-04-06 |
| **hCoV-19/Germany/SN-ChVir-W1147-4309/2020** | Germany | Saxony | EPI_ISL_754048 | 2020-04-06 |
| **hCoV-19/Germany/BE-ChVir8329/2020** | Germany | Berlin | EPI_ISL_729584 | 2020-08-01 |
| **hCoV-19/Germany/BE-ChVir-D1930-8296/2020** | Germany | Berlin | EPI_ISL_753873 | 2020-10-10 |
| **hCoV-19/Germany/BE-ChVir-D2017-8214/2020** | Germany | Berlin | EPI_ISL_753902 | 2020-10-19 |
| **hCoV-19/Germany/BE-ChVir-D1972-8263/2020** | Germany | Berlin | EPI_ISL_753815 | 2020-10-05 |
| **hCoV-19/Germany/BE-ChVir-W1725-8201/2020** | Germany | Berlin | EPI_ISL_754012 | 2020-08-20 |
| **hCoV-19/Germany/ST-MD144/2020** | Germany | Saxony-Anhalt | EPI_ISL_864566 | 2020-10-22 |
| **hCoV-19/Germany/BE-ChVir-D1919-4432/2020** | Germany | Berlin | EPI_ISL_753865 | 2020-10-04 |
| **hCoV-19/Germany/BE-ChVir-W1729-5875/2020** | Germany | Berlin | EPI_ISL_754015 | 2020-08-17 |
| **hCoV-19/Germany/BE-ChVir9031/2020** | Germany | Berlin | EPI_ISL_729409 | 2020-09-20 |
| **hCoV-19/Germany/BE-ChVir8990/2020** | Germany | Berlin | EPI_ISL_729365 | 2020-09-22 |
| **hCoV-19/Germany/BE-ChVir9034/2020** | Germany | Berlin | EPI_ISL_729607 | 2020-09-20 |
| **hCoV-19/Germany/BE-ChVir-W1286-4312/2020** | Germany | Berlin | EPI_ISL_753759 | 2020-04-18 |
| **hCoV-19/Germany/BE-ChVir-W1140-8100/2020** | Germany | Berlin | EPI_ISL_753941 | 2020-04-07 |
| **hCoV-19/Germany/BE-RKI-Z-0021/2020** | Germany | Berlin | EPI_ISL_483154 | 2020-04-27 |
| **hCoV-19/Germany/BE-RKI-Z-0022/2020** | Germany | Berlin | EPI_ISL_483155 | 2020-04-27 |
| **hCoV-19/Germany/BB-ChVir-W1646-6734/2020** | Germany | Brandenburg | EPI_ISL_753858 | 2020-08-05 |
| **hCoV-19/Germany/BE-ChVir-W1703-6771/2020** | Germany | Berlin | EPI_ISL_753995 | 2020-08-19 |
| **hCoV-19/Germany/BE-ChVir9006/2020** | Germany | Berlin | EPI_ISL_729602 | 2020-09-22 |
| **hCoV-19/Germany/BE-ChVir9025/2020** | Germany | Berlin | EPI_ISL_729382 | 2020-09-19 |
| **hCoV-19/Germany/BE-ChVir-W1636-6724/2020** | Germany | Berlin | EPI_ISL_753703 | 2020-07-30 |
| **hCoV-19/Germany/BE-ChVir-W1637-6714/2020** | Germany | Berlin | EPI_ISL_753974 | 2020-08-02 |
| **hCoV-19/Germany/BE-ChVir-W1699-8236/2020** | Germany | Berlin | EPI_ISL_753708 | 2020-07-29 |
| **hCoV-19/Germany/BE-ChVir-W1709-8206/2020** | Germany | Berlin | EPI_ISL_753999 | 2020-08-10 |
| **hCoV-19/Germany/BE-ChVir-D1927-7462/2020** | Germany | Berlin | EPI_ISL_753871 | 2020-10-09 |
| **hCoV-19/Germany/BE-ChVir-W1316-2147/2020** | Germany | Berlin | EPI_ISL_753752 | 2020-04-16 |
| **hCoV-19/Germany/BE-ChVir-W1639-6740/2020** | Germany | Berlin | EPI_ISL_753976 | 2020-08-03 |
| **hCoV-19/Germany/BE-ChVir-D1935-5099/2020** | Germany | Berlin | EPI_ISL_753875 | 2020-10-10 |
| **hCoV-19/Germany/BE-ChVir-D2027-8242/2020** | Germany | Berlin | EPI_ISL_753910 | 2020-10-14 |
| **hCoV-19/Germany/BE-ChVir9002/2020** | Germany | Berlin | EPI_ISL_729356 | 2020-09-21 |
| **hCoV-19/Germany/BE-ChVir-W1708-6787/2020** | Germany | Berlin | EPI_ISL_753998 | 2020-08-05 |
| **hCoV-19/Germany/BE-ChVir-W1710-6707/2020** | Germany | Berlin | EPI_ISL_754000 | 2020-08-06 |
| **hCoV-19/Germany/BE-ChVir-D2024-8294/2020** | Germany | Berlin | EPI_ISL_753907 | 2020-09-28 |
| **hCoV-19/Germany/BE-ChVir-D1923-8213/2020** | Germany | Berlin | EPI_ISL_753868 | 2020-10-05 |
| **hCoV-19/Germany/BE-ChVir-D2036-8292/2020** | Germany | Berlin | EPI_ISL_753914 | 2020-10-08 |
| **hCoV-19/Germany/BE-ChVir-D1987-4459/2020** | Germany | Berlin | EPI_ISL_753773 | 2020-10-02 |
| **hCoV-19/Germany/BE-ChVir-D1983-4479/2020** | Germany | Berlin | EPI_ISL_753819 | 2020-09-26 |
| **hCoV-19/Germany/TH-IIMK-CaSe-28/2020** | Germany | Thuringia | EPI_ISL_763093 | 2020-04-03 |
| **hCoV-19/Germany/BE-RKI-Z-0023/2020** | Germany | Berlin | EPI_ISL_483156 | 2020-04-27 |
| **hCoV-19/Germany/BE-ChVir8973/2020** | Germany | Berlin | EPI_ISL_729388 | 2020-09-18 |
| **hCoV-19/Germany/BE-ChVir-W1711-8245/2020** | Germany | Berlin | EPI_ISL_754001 | 2020-08-08 |
| **hCoV-19/Germany/BE-ChVir-D1924-8211/2020** | Germany | Berlin | EPI_ISL_753869 | 2020-10-09 |
| **hCoV-19/Germany/BE-ChVir-D2008-8215/2020** | Germany | Berlin | EPI_ISL_753897 | 2020-10-15 |
| **hCoV-19/Germany/TH-IIMK-CaSe-22/2020** | Germany | Thuringia | EPI_ISL_763087 | 2020-03-19 |
| **hCoV-19/Germany/TH-IIMK-CaSe-21/2020** | Germany | Thuringia | EPI_ISL_763086 | 2020-03-19 |
| **hCoV-19/Germany/TH-IIMK-CaSe-24/2020** | Germany | Thuringia | EPI_ISL_763089 | 2020-03-25 |
| **hCoV-19/Germany/TH-IIMK-CaSe-25/2020** | Germany | Thuringia | EPI_ISL_763090 | 2020-03-25 |
| **hCoV-19/Germany/BE-ChVir-W1336-5617/2020** | Germany | Berlin | EPI_ISL_753954 | 2020-05-05 |
| **hCoV-19/Germany/ST-MD207/2020** | Germany | Saxony-Anhalt | EPI_ISL_884278 | 2020-12-18 |
| **hCoV-19/Germany/ST-MD248/2020** | Germany | Saxony-Anhalt | EPI_ISL_884279 | 2020-12-30 |
| **hCoV-19/Germany/BB-RKI-NP-0057/2020** | Germany | Brandenburg | EPI_ISL_763056 | 2020-12-14 |
| **hCoV-19/Germany/BB-RKI-NP-0068/2020** | Germany | Brandenburg | EPI_ISL_763053 | 2020-12-16 |
| **hCoV-19/Germany/BB-RKI-NP-0058/2020** | Germany | Brandenburg | EPI_ISL_763055 | 2020-12-14 |
| **hCoV-19/Germany/ST-MD227/2020** | Germany | Saxony-Anhalt | EPI_ISL_884285 | 2020-12-25 |
| **hCoV-19/Germany/SN-RKI-NP-0013/2020** | Germany | Saxony | EPI_ISL_763021 | 2020-12-03 |
| **hCoV-19/Germany/BE-ChVir-D2048-8293/2020** | Germany | Berlin | EPI_ISL_753710 | 2020-10-06 |
| **hCoV-19/Germany/BE-ChVir-D1975-4407/2020** | Germany | Berlin | EPI_ISL_753737 | 2020-10-06 |
| **hCoV-19/Germany/ST-MD179/2020** | Germany | Saxony-Anhalt | EPI_ISL_864574 | 2020-12-15 |
| **hCoV-19/Germany/TH-RKI-NP-0012/2020** | Germany | Thuringia | EPI_ISL_763008 | 2020-12-03 |
| **hCoV-19/Germany/TH-RKI-NP-0021/2020** | Germany | Thuringia | EPI_ISL_763006 | 2020-12-07 |
| **hCoV-19/Germany/TH-RKI-NP-0054/2020** | Germany | Thuringia | EPI_ISL_763002 | 2020-12-14 |
| **hCoV-19/Germany/TH-IIMK-CaSe-26/2020** | Germany | Thuringia | EPI_ISL_763091 | 2020-03-27 |
| **hCoV-19/Germany/BE-ChVir-W1341-4391/2020** | Germany | Berlin | EPI_ISL_753761 | 2020-04-10 |
| **hCoV-19/Germany/TH-IIMK-CaSe-27/2020** | Germany | Thuringia | EPI_ISL_763092 | 2020-04-01 |
| **hCoV-19/Germany/BE-ChVir-W1060-4393/2020** | Germany | Berlin | EPI_ISL_753928 | 2020-03-30 |
| **hCoV-19/Germany/BE-ChVir-W1395-5720/2020** | Germany | Berlin | EPI_ISL_753775 | 2020-05-11 |
| **hCoV-19/Germany/BE-ChVir-D1911-8207/2020** | Germany | Berlin | EPI_ISL_753860 | 2020-09-29 |
| **hCoV-19/Germany/BE-ChVir-D1914-8251/2020** | Germany | Berlin | EPI_ISL_753862 | 2020-10-05 |
| **hCoV-19/Germany/BE-ChVir-D1943-5032/2020** | Germany | Berlin | EPI_ISL_753879 | 2020-10-01 |
| **hCoV-19/Germany/BE-ChVir-D1945-8218/2020** | Germany | Berlin | EPI_ISL_753880 | 2020-10-02 |
| **hCoV-19/Germany/SN-RKI-Z-0038/2020** | Germany | Saxony | EPI_ISL_825167 | 2020-12-23 |
| **hCoV-19/Germany/BE-ChVir8981/2020** | Germany | Berlin | EPI_ISL_729413 | 2020-09-22 |
| **hCoV-19/Germany/BE-ChVir8972/2020** | Germany | Berlin | EPI_ISL_729346 | 2020-09-18 |
| **hCoV-19/Germany/BE-ChVir-W1537-4361/2020** | Germany | Berlin | EPI_ISL_753730 | 2020-05-18 |
| **hCoV-19/Germany/BE-ChVir-W1439-5711/2020** | Germany | Berlin | EPI_ISL_753967 | 2020-06-20 |
| **hCoV-19/Germany/BE-ChVir-W2127-8203/2020** | Germany | Berlin | EPI_ISL_754192 | 2020-10-07 |
| **hCoV-19/Germany/BB-ChVir-D1985-4492/2020** | Germany | Brandenburg | EPI_ISL_754185 | 2020-09-28 |
| **hCoV-19/Germany/BE-ChVir-D2030-4443/2020** | Germany | Berlin | EPI_ISL_753715 | 2020-10-15 |
| **hCoV-19/Germany/BE-ChVir-D2019-4498/2020** | Germany | Berlin | EPI_ISL_754194 | 2020-10-19 |
| **hCoV-19/Germany/BB-ChVir-W1533-4338/2020** | Germany | Berlin | EPI_ISL_753712 | 2020-05-11 |
| **hCoV-19/Germany/BE-ChVir-W1323-5723/2020** | Germany | Berlin | EPI_ISL_753824 | 2020-05-11 |
| **hCoV-19/Germany/BE-ChVir-W1724-8220/2020** | Germany | Berlin | EPI_ISL_753742 | 2020-08-11 |
| **hCoV-19/Germany/BE-ChVir7622/2020** | Germany | Berlin | EPI_ISL_729556 | 2020-06-05 |
| **hCoV-19/Germany/ST-MD260/2020** | Germany | Saxony-Anhalt | EPI_ISL_884290 | 2020-12-28 |
| **hCoV-19/Germany/ST-MD203/2020** | Germany | Saxony-Anhalt | EPI_ISL_884281 | 2020-12-21 |
| **hCoV-19/Germany/ST-MD259/2020** | Germany | Saxony-Anhalt | EPI_ISL_884289 | 2020-12-28 |
